# ﻿*Nothotytthonyx*, a new genus of Malthininae (Coleoptera, Cantharidae) from mid-Cretaceous amber of northern Myanmar

**DOI:** 10.3897/zookeys.1092.81701

**Published:** 2022-04-04

**Authors:** Yan-Da Li, Gabriel Biffi, Robin Kundrata, Di-Ying Huang, Chen-Yang Cai

**Affiliations:** 1 State Key Laboratory of Palaeobiology and Stratigraphy, Nanjing Institute of Geology and Palaeontology, and Centre for Excellence in Life and Paleoenvironment, Chinese Academy of Sciences, Nanjing 210008, China Centre for Excellence in Life and Paleoenvironment, Chinese Academy of Sciences Nanjing China; 2 School of Earth Sciences, University of Bristol, Life Sciences Building, Tyndall Avenue, Bristol BS8 1TQ, UK University of Bristol Bristol United Kingdom; 3 Museu de Zoologia, Universidade de São Paulo, Av. Nazaré, 481– Ipiranga, 04263-000, São Paulo, SP, Brazil Universidade de São Paulo São Paulo Brazil; 4 Department of Zoology, Faculty of Science, Palacký University, 77900 Olomouc, Czech Republic Palacký University Olomouc Czech Republic

**Keywords:** Burmese amber, Cretaceous, fossil, paleontology, soft-bodied Elateroidea, soldier beetles, systematics

## Abstract

A new fossil genus and species of Cantharidae, *Nothotytthonyxserratus* Li, Biffi, Kundrata & Cai **gen. et sp. nov.**, is reported from mid-Cretaceous Burmese amber. The new species is tentatively attributed to the extant subfamily Malthininae based on a combination of characters, including the symmetrical apical maxillary palpomeres, shortened elytra, pronotum with arched margins and well-defined borders, tibiae with apical spurs, and tarsal claws simple, although its well-developed gonostyli are atypical in Malthininae. The discovery of *Nothotytthonyx* also suggests a possible Gondwanan origin for Malthininae.

## ﻿Introduction

Cantharidae is a diverse group among the soft-bodied Elateroidea, with over 5000 species distributed worldwide (Ramsdale 2010). Cantharid adults are highly active, and may feed on foliage-frequenting invertebrates, nectar or pollen (Crowson 1972; Ramsdale 2010). Both larvae and adults of cantharids have paired lateral glandular pores for chemical defense against predators, although the pores may sometimes be inconspicuous and hard to determine. The family appears to be closely related to the elaterid-lampyroid group. However, its accurate position remains unsettled, as several recent phylogenomic studies have produced inconsistent results (Zhang et al. 2018; McKenna et al. 2019; Douglas et al. 2021; Cai et al. 2022). Brancucci (1980) conducted a comprehensive study on the taxonomy of Cantharidae, and divided it into five subfamilies, namely Cantharinae, Chauliognathinae, Dysmorphocerinae, Silinae and Malthininae. The relationships among the subfamilies varied in different studies and were not well understood (e.g., Brancucci 1980; Kundrata et al. 2014; McKenna et al. 2015, 2019; Zhang et al. 2018; Hsiao et al. 2021; Cai et al. 2022).

Records of fossil cantharids are relatively abundant, especially in amber deposits. More than 80 species have been reported from Eocene Baltic amber (e.g., Fanti and Kupryjanowicz 2017; Fanti and Damgaard 2020; Fanti 2021). Additional cantharids have been described from Early Cretaceous Spanish amber (Peris and Fanti 2018), Late Cretaceous Agdzhakend amber (Kazantsev and Perkovsky 2019a), Eocene Rovno amber (Kazantsev and Perkovsky 2020), Eocene Sakhalinian amber (Kazantsev and Perkovsky 2019b), and Miocene Dominican amber (Fanti and Damgaard 2019; Fanti and Pankowski 2021). From the fossiliferous mid-Cretaceous Burmese amber, about 20 species have been described in nine genera (e.g., Hsiao et al. 2021; Yang et al. 2021). Most cantharids from Burmese amber were originally assigned to Cantharinae diagnosed by, for example, securiform apical maxillary palpomeres, unmodified pronotal margins and well-developed elytra concealing the wings and abdomen. However, the subfamilial placement of at least some of the genera (*Ornatomalthinus* Poinar & Fanti and *Sanaungulus* Fanti et al.) is controversial due to their conflicting diagnostic characters (Fanti 2018; Hsiao and Huang 2018; Hsiao et al. 2021). In the morphology-based phylogenetic analysis by Hsiao et al. (2021), such group of genera was retrieved forming the “Burmite Cantharinae” clade, as sister to Silinae, and their placement within Cantharinae was rejected. In the same work, *Archaeomalthodes* Hsiao et al., originally placed in Malthininae (Hsiao et al, 2017), was suggested as a member of Dysmorphocerinae (Hsiao et al. 2021). Here, we describe a new fossil cantharid from Burmese amber, and tentatively assign it to subfamily Malthininae.

## ﻿Materials and methods

**Figure 1.**
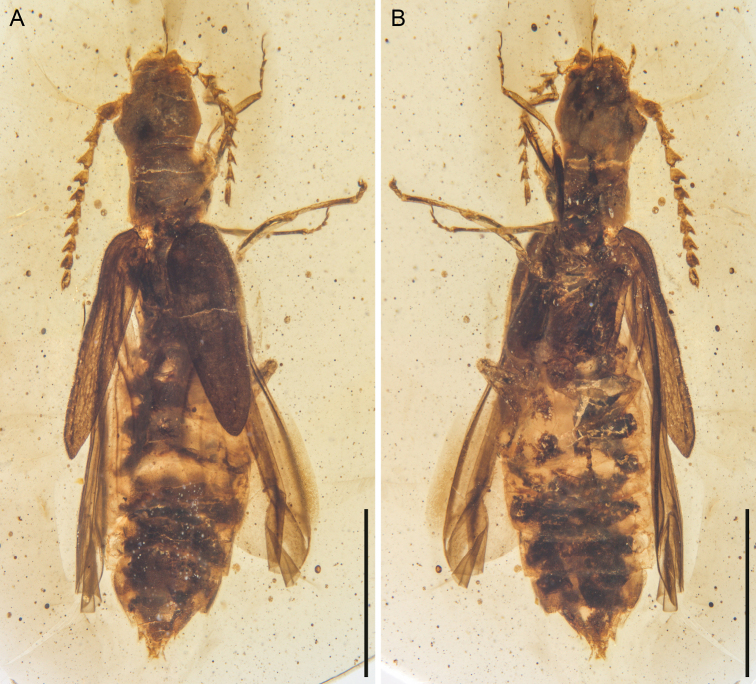
General habitus of *Nothotytthonyxserratus* Li, Biffi, Kundrata & Cai sp. nov., holotype, NIGP179427, under incident light **A** dorsal view **B** ventral view. Scale bars: 1.5 mm.

**Figure 2.**
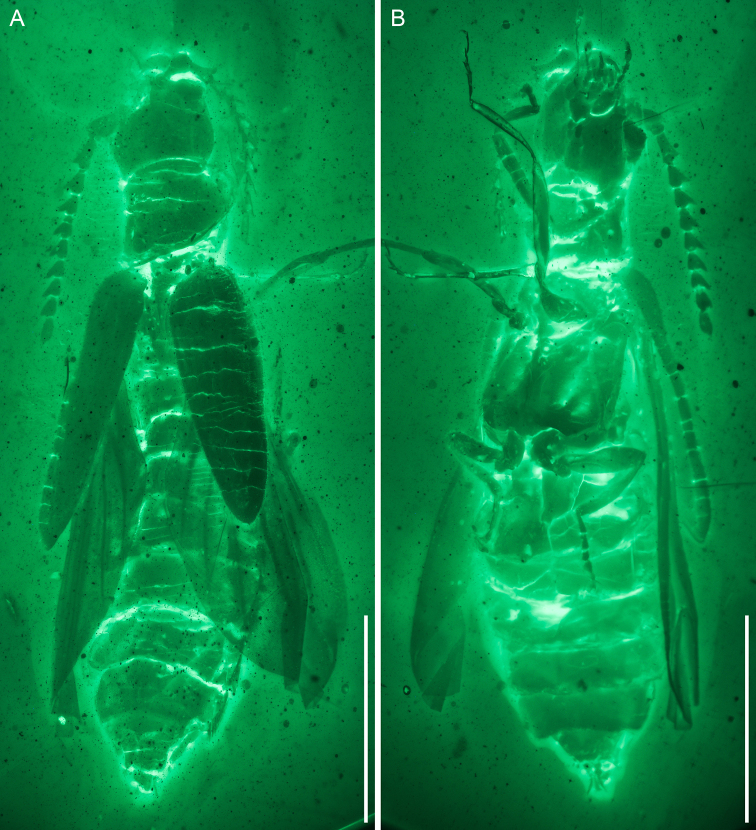
General habitus of *Nothotytthonyxserratus* Li, Biffi, Kundrata & Cai sp. nov., holotype, NIGP179427, under widefield fluorescence **A** dorsal view. **B** ventral view. Scale bars: 1.5 mm.

**Figure 3.**
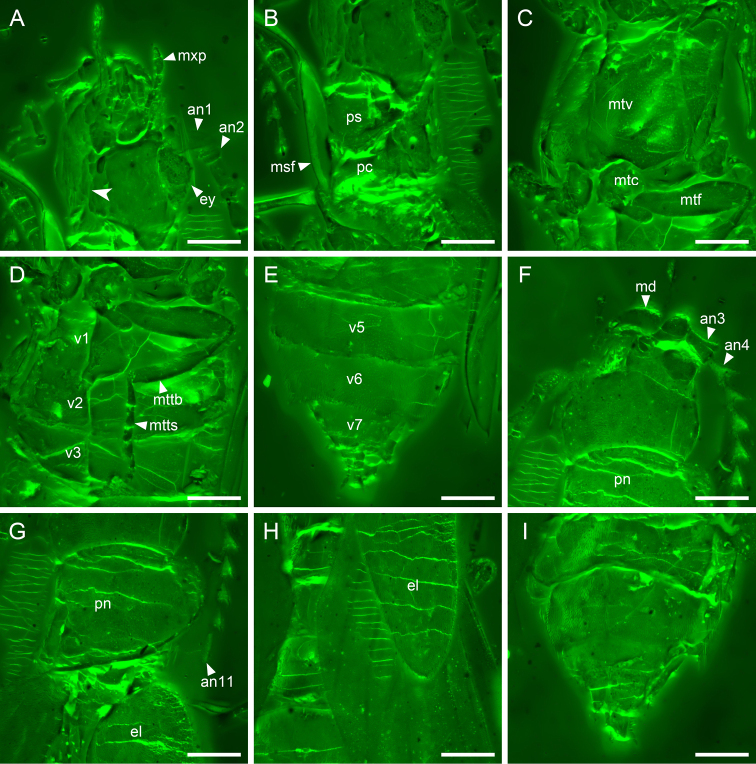
Details of *Nothotytthonyxserratus* Li, Biffi, Kundrata & Cai sp. nov., holotype, NIGP179427, under confocal microscopy **A** head, ventral view, showing the confluent gular suture (arrowhead) **B** prothorax, ventral view **C** metathorax, ventral view **D** abdominal base, ventral view **E** abdominal apex, ventral view **F** head, dorsal view **G** prothorax, dorsal view **H** elytral base, dorsal view **H** abdominal apex, dorsal view. Abbreviations: an1–11, antennomeres 1–11; el, elytron; ey, compound eye; md, mandible; msf, mesofemur; mtc, metacoxa; mttb, metatibia; mtts, metatarsus; mtv, metaventrite; mxp, maxillary palp; pc, procoxa; pn, pronotum; ps, prosternum; v1–7, ventrites 1–7. Scale bars: 300 μm.

**Figure 4.**
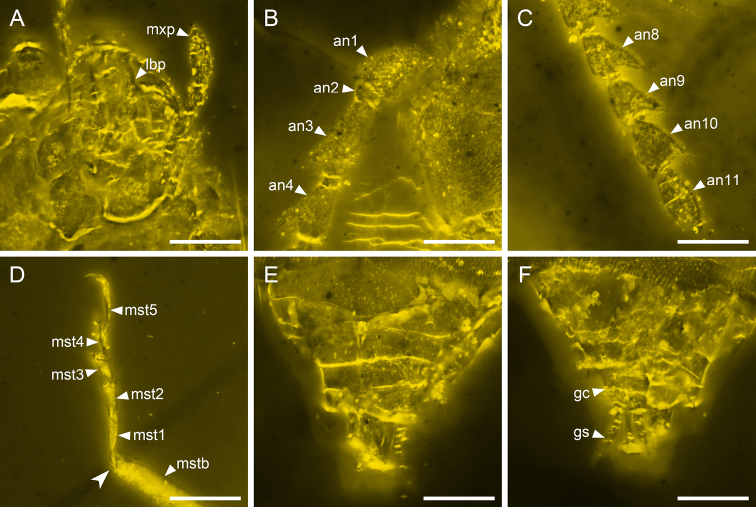
Details of *Nothotytthonyxserratus* Li, Biffi, Kundrata & Cai sp. nov., holotype, NIGP179427, under confocal microscopy **A** mouthparts, ventral view **B** antennal base, dorsal view **C** antennal apex **D** mid leg, showing the two weak tibial spurs (arrowhead) **E** abdominal apex, dorsal view **F** ovipositor, ventral view. Abbreviations: an1–11, antennomeres 1–11; gc, gonocoxite; gs, gonostylus; lbp, labial palp; mst1–5, mesotarsomeres 1–5; mstb, mesotibia; mxp, maxillary palp. Scale bars: 200 μm.

**Figure 5.**
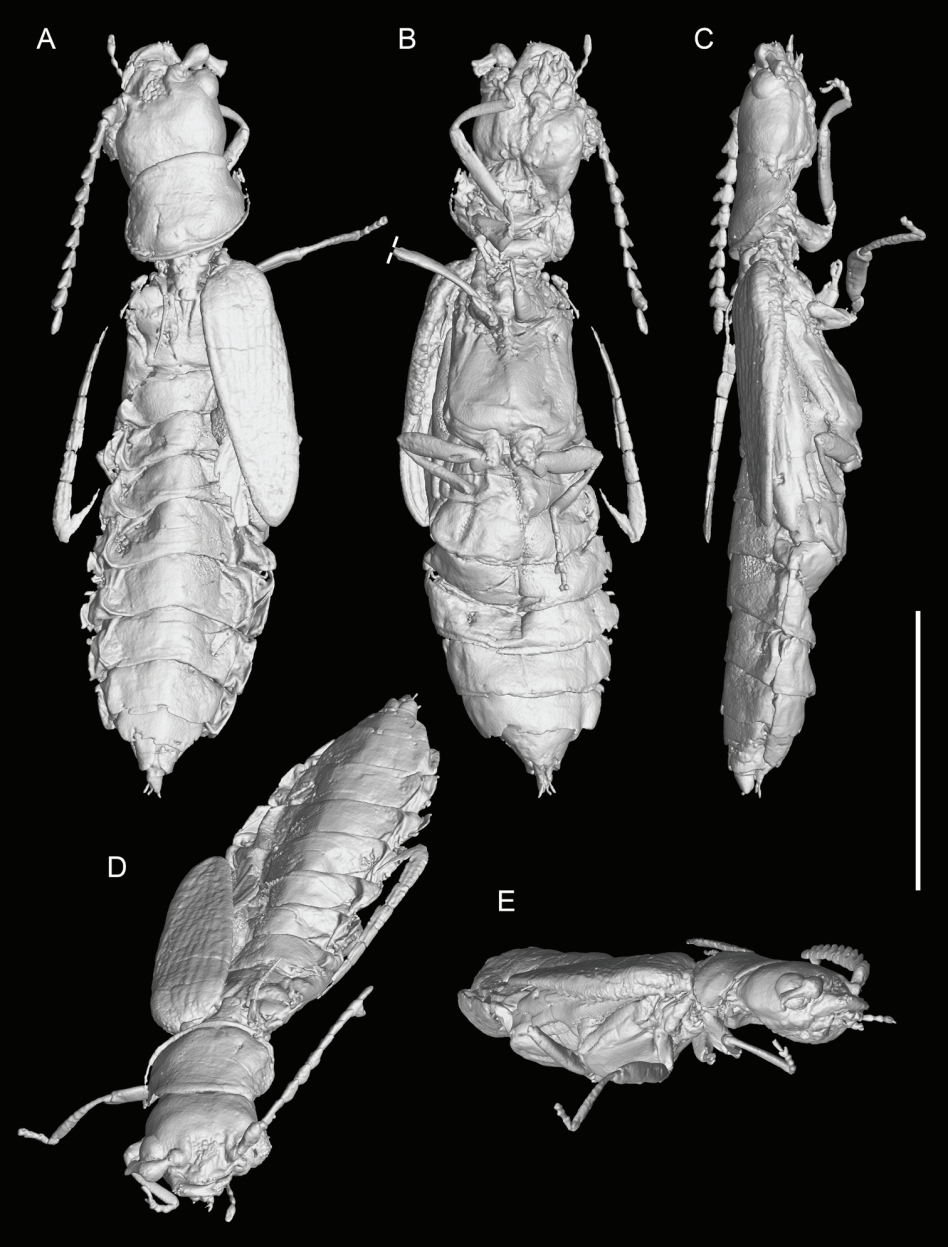
X-ray microtomographic reconstruction of *Nothotytthonyxserratus* Li, Biffi, Kundrata & Cai sp. nov., holotype, NIGP179427 **A** dorsal view **B** ventral view **C** lateral view **D** anterodorsal view **E** anterolateral view. Scale bar: 2 mm.

The Burmese amber specimen studied herein (Figs 1–5) originated from an amber mine near Noije Bum (26°20'N, 96°36'E), Hukawng Valley, Kachin State, northern Myanmar. Jewellery-grade Burmese amber specimens are commonly carried and sold legally in Ruili, Dehong Prefecture on the border between China and Myanmar. The specimen in this study was purchased in late 2016, and is permanently deposited in the Nanjing Institute of Geology and Palaeontology (**NIGP**), Chinese Academy of Sciences, Nanjing, China. The amber piece was trimmed with a small table saw, ground with emery paper of different grit sizes, and finally polished with polishing powder.

Photographs under incident light were taken with a Zeiss Discovery V20 stereo microscope. Widefield fluorescence images were captured with a Zeiss Axio Imager 2 light microscope combined with a fluorescence imaging system. Confocal images were obtained with a Zeiss LSM710 confocal laser scanning microscope, using the 488 nm (Argon) or 561 nm (DPSS 561-10) laser excitation lines (Fu et al. 2021). Images under incident light and widefield fluorescence were stacked in Helicon Focus 7.0.2 or Zerene Stacker 1.04. Confocal images were stacked with Helicon Focus 7.0.2 and Adobe Photoshop CC. Microtomographic data were obtained with a Zeiss Xradia 520 Versa 3D X-ray microscope at the micro-CT laboratory of NIGP and analyzed in VGStudio MAX 3.0. Scanning parameters were as follows: isotropic voxel size, 6.1511 μm; power, 4 W; acceleration voltage, 50 kV; exposure time, 1.5 s; projections, 2401. Images were further processed in Adobe Photoshop CC to adjust brightness and contrast.

## ﻿Data availability

The original confocal and micro-CT data are available in Zenodo repository (https://doi.org/10.5281/zenodo.6336149).

## ﻿Systematic paleontology

### ﻿Order Coleoptera Linnaeus, 1758

**Superfamily Elateroidea Leach, 1815**

**Family Cantharidae Imhoff, 1856**

#### Subfamily Malthininae Kiesenwetter, 1852

##### ﻿Genus Nothotytthonyx

Taxon classificationAnimaliaColeopteraCantharidae

Li, Biffi, Kundrata & Cai, gen. nov.

1827AB42-C386-53D4-9506-6AA297959899

http://zoobank.org/A56DECAD-2C71-4822-B48C-690DD67B4C3E

###### Type species.

*Nothotytthonyxserratus* sp. nov.

###### Etymology.

The generic name is derived from the Greek “*nothos*”, false, and the generic name *Tytthonyx* LeConte. The name is masculine in gender.

###### Diagnosis.

Antennae strongly serrate (Figs 3F, 4C). Mandibles with a prominent tooth on incisor edge (Fig. 5D). Apical maxillary palpomere symmetrical, fusiform (Fig. 4A). Gular sutures confluent (Figs 3A, 5B). Elytra shortened; surface somewhat punctate. Tibial spurs present (Fig. 4D). Gonostyli well developed (Fig. 4F).

##### ﻿ Nothotytthonyx serratus

Taxon classificationAnimaliaColeopteraCantharidae

 Li, Biffi, Kundrata & Cai, sp. nov.

60DC9501-CDB1-54D5-87A2-03211C416C54

http://zoobank.org/44E1AFDB-31BC-4B43-B8FA-D3F566934E3F

Figs 1 , 2 , 3 , 4 , 5

###### Material.

***Holotype***, NIGP179427, female.

###### Etymology.

The specific name refers to its distinctly serrate antennae.

###### Locality and horizon.

Amber mine located near Noije Bum Village (26°20'N, 96°36'E), Tanai Township, Myitkyina District, Kachin State, Myanmar; unnamed horizon, mid-Cretaceous, Upper Albian to Lower Cenomanian.

###### Diagnosis.

As for the genus.

###### Description.

**Adult female.** Body weakly sclerotized, elongate, about 5.3 mm long, 1.4 mm wide (widest across abdomen).

***Head*** (Fig. 3A,F) fully exposed, prognathous, subquadrate, weakly narrowed posteriorly, including eyes almost as wide as pronotum; dorsal surface flat, without protuberance or depression. Compound eyes moderately large and weakly protruding, finely facetted, without interfacetal setae. Antennal insertions located anteriorly, dorsally exposed, separated by approximately the length of antennomere 1. Subantennal grooves absent. Antennae (Fig. 4B,C) with 11 antennomeres; antennomere 1 moderately broad; antennomeres 2 short; antennomere 3–10 distinctly serrate. Mandibles with one prominent tooth on incisor edge (Fig. 5D). Apical maxillary and labial palpomeres elongate, symmetrical, fusiform, not unequally expanded, apex acute (Fig. 4A). Gular sutures confluent (Figs 3A, 5B).

Pronotal disc (Fig. 3G) transverse; anterior and posterior angles broadly rounded; lateral and posterior margins clearly bordered. Elytra (Fig. 3H) relatively short, covering only about half of posterior body; surface somewhat punctate. Procoxae (Fig. 5B) conical, well projecting, contiguous. Mesocoxae (Fig. 5B) conical, well projecting, narrowly separated. Metaventrite (Fig. 3C) large, with distinct discrimen and metakatepisternal suture. Metacoxae (Fig. 3C) transverse, almost contiguous.

***Legs*** slender. Trochanters obliquely articulated to femoral bases. Tibiae with weak spurs (at least as seen on left mesotibia; Fig. 4D). Tarsal formula 5–5–5; tarsomere 4 ventrally bilobed (Figs 4D, 5E). Claws simple.

***Abdomen*** with seven free ventrites. Gonostyli well developed (Fig. 4F).

## ﻿Discussion

Within soft-bodied elateroids, *Nothotytthonyx* is firmly placed in Cantharidae, primarily based on the fully exposed prognathous head (Fig. 3A,F), 11-segmented antennae (Fig. 1), and ventrally bilobed tarsomere 4 (Figs 4D, 5E).

The current classification of Cantharidae into five subfamilies is solely based on extant species (Brancucci 1980). However, there are no incontestable diagnostic characters for most subfamilies (except for Chauliognathinae). Many characters may have evolved independently in separate subfamilies, and some characters used for diagnosis may be absent in certain lineages within a subfamily, which hampers the precise systematic placement of some genera (e.g., *Tytthonyx*). In the case of fossils, this problem is aggravated by the impossibility of observation of important characters, especially the genitalia and wing venation, leading to the conflicting hypotheses of placement (e.g., Fanti 2018; Hsiao and Huang 2018; Hsiao et al. 2021). For instance, Malthininae and a few members of Dysmorphocerinae have radially symmetrical apical maxillary palpomeres, while in other subfamilies and most of Dysmorphocerinae the apical maxillary palpomeres are securiform, except for *Tytthonyx*, currently classified as *incertae sedis* in Silinae. Dysmorphocerinae, however, generally have a wide pronotum and complete elytra. The elytra are reduced in most of Malthininae genera, although this feature is also present in species in most other subfamilies (e.g., Chauliognathinae: *Ichthyurus* Westwood, *Lobetus* Kiesenwetter; Cantharinae: some *Lycocerus* Gorham; Silinae: some *Polemius* LeConte, *Brachysilidius* Pic).

*Nothotytthonyx* is herein tentatively assigned to the subfamily Malthininae by a combination of characters, such as the radially symmetrical apical maxillary palpomeres, shortened elytra, pronotum with arched margins and well-defined borders, tibiae with apical spurs, and tarsal claws simple. However, the ovipositor with long gonostyli of *Nothotytthonyx* seems to be quite aberrant in Malthininae. No extant species of Malthininae (and Dysmorphocerinae and Silinae) has long and clearly defined styli. According to Brancucci (1980), the well-defined coxites and styli are the “primitive form”, and they are typical of the subfamily Cantharinae. In Malthininae, the styli are indistinct; according to Brancucci, they are either extremely reduced or, most probably, solidly fused to the coxites, and correspond to the pubescent area of the coxites.

Within Malthininae, Malthinini have confluent or almost confluent gular sutures, while Malthodini and Malchinini have separated gular sutures (Brancucci 1980). Besides, there is a prominent tooth on the incisor edge of mandibles in Malthinini, while in Malthodini and Malchinini the mandibles are armed with a weak tooth, with a row of small teeth, or simple (without teeth) (Brancucci 1980). *Nothotytthonyx* differs from genera in the Malthodini and Malchinini in having confluent gular sutures and mandibles with a prominent tooth on the incisor edge. Yet, *Nothotytthonyx* is distinctive among genera in Malthinini for having strongly serrate antennae. Most genera in Malthinini have filiform or weakly serrate antennae, and *Paramalthinus* Brancucci has pectinate antennae (even though the antennae of *Paramalthinus* are pectinate, its antennomere bodies are rather elongate). *Nothotytthonyx* is different from other genera in Malthinini additionally in the combination of the moderately shortened elytra, clearly confluent gular sutures, presence of tibial spurs, and unelongated metacoxae (Brancucci 1980; Fanti and Castiglione 2017; Fanti and Vitali 2017).

It is notable that *Nothotytthonyx* is somewhat similar to *Tytthonyx*. This genus shares characters with both Malthininae (e.g., mandibles with retinaculum, radially symmetrical apical maxillary palpomeres, the shape of pronotum, reduced elytra and wing venation) and Silinae (e.g., the structures of terminal ventrites and tergites and the aedeagus). *Tytthonyx* has been kept in its own tribe Tytthonyxini as *incertae sedis* in Silinae (Brancucci 1980); however, in a recent morphology-based phylogenetic analysis, *Tytthonyx* was revealed as the sister group of Malthininae (Hsiao et al. 2021). *Nothotytthonyx* shares with *Tytthonyx* a similar habitus, symmetrical apical maxillary palpomeres, and shortened elytra. In some species of Tytthonyx (subgenus Tytthonyx), the antennae are also distinctly serrate. *Nothotytthonyx* nevertheless differs from *Tytthonyx* in the confluent gular sutures (separated in *Tytthonyx*) and the structure of abdomen (gonostyli absent in *Tytthonyx*; Brancucci 1980).

Malthininae today generally have a Holarctic (Laurasian) distribution, with only limited fauna known from Gondwanan parts of the World, whereas Dysmorphocerinae have a strictly Gondwanan distribution. Although *Archaeomalthodes* from Burmese amber was once classified in Malthininae (Hsiao et al. 2017), it was later revised as a member of Dysmorphocerinae (Hsiao et al. 2021). Thus, as the first fossil of Malthininae from Burmese amber and also from the Mesozoic Era, *Nothotytthonyx* has important biogeographical implications. If we accept that the Burmese amber, which comes from the mines located on the West Burma Block, is of a Gondwanan origin (Poinar 2019), then our current discovery indicates that Malthininae were present in Gondwanan lands in the Mesozoic, and taking into consideration that they are not known from any northern-hemisphere Mesozoic deposits, they may have originated in the south, and only later dispersed to north where they greatly diversified and survived until now whereas they became rare in the south. However, this hypothesis will need to be tested in future.

## Supplementary Material

XML Treatment for Nothotytthonyx

XML Treatment for Nothotytthonyx serratus
